# A comparative evaluation of nature-inspired algorithms for feature selection problems

**DOI:** 10.1016/j.heliyon.2023.e23571

**Published:** 2023-12-12

**Authors:** Mariappan Premalatha, Murugan Jayasudha, Robert Čep, Jayaraju Priyadarshini, Kanak Kalita, Prasenjit Chatterjee

**Affiliations:** a School of Computer Science & Engineering, Vellore Institute of Technology, Chennai 600 127, India; b Department of Machining, Assembly and Engineering Metrology, Faculty of Mechanical Engineering, VSB-Technical University of Ostrava, 17. Listopadu 2172/15, 708 00 Ostrava, Czech Republic; c University Centre for Research & Development, Chandigarh University, Mohali, 140413, India; d Chief Research Fellow, Faculty of Civil Engineering, Institute of Sustainable Construction, Laboratory of Smart Building Systems, Vilnius Gediminas Technical University, Vilnius, Lithuania

**Keywords:** Optimization, Non-traditional algorithms, Feature reduction, KNN, Algorithms, Metaheuristics

## Abstract

Feature selection is a critical component of machine learning and data mining which addresses challenges like irrelevance, noise, redundancy in large-scale data etc., which often result in the curse of dimensionality. This study employs a K-nearest neighbour wrapper to implement feature selection using six nature-inspired algorithms, derived from human behaviour and mammal-inspired techniques. Evaluated on six real-world datasets, the study aims to compare the performance of these algorithms in terms of accuracy, feature count, fitness, convergence and computational cost. The findings underscore the efficacy of the Human Learning Optimization, Poor and Rich Optimization and Grey Wolf Optimizer algorithms across multiple performance metrics. For instance, for mean fitness, Human Learning Optimization outperforms the others, followed by Poor and Rich Optimization and Harmony Search. The study suggests the potential of human-inspired algorithms, particularly Poor and Rich Optimization, in robust feature selection without compromising classification accuracy.

## 1 Introduction

Feature selection is an integral part of machine learning applications, serving as a crucial step to reduce data dimensionality. Such a reduction is essential, particularly in the current era with overwhelming data flow. One major challenge faced is the presence of redundant or irrelevant data features that may not be suitable for specific applications [1]. Traditional approaches to address this have involved conventional optimization methods like Hill-climbing or gradient-based optimization methods. However, there has been a shift in interest towards more sophisticated optimization techniques, known as metaheuristics [2].

The term ‘metaheuristic’ was formalized in the mid-nineties and is rooted in the words ‘meta’ and ‘heuristic’ [3]. While ‘meta’ indicates beyond the normal limit, ‘heuristic’ signifies self-learning or discovery [4]. Metaheuristic techniques play a pivotal role in machine learning systems by carrying out three distinct functions: exploration, exploitation, and optimum solution determination. A noticeable aspect of these techniques is their inspiration, which often comes from natural phenomena like the behaviors of specific species or social or physical events. Consequently, a vast number of optimization metaheuristic models have been developed [5,6].

Despite the potential of metaheuristics, there exist challenges in determining which method is optimal for feature selection. Each metaheuristic has its strengths and shortcomings, and their applicability to feature selection varies. This discrepancy forms the crux of our investigation. We aim to provide clarity by comparing the effectiveness of three human behaviour-inspired algorithms (namely Harmony Search (HS) [7], Human Learning Optimization (HLO) [8], and Poor and Rich Optimization (PRO)) [9] against three mammal-inspired algorithms (Bat Algorithm (BA) [10], Grey Wolf Optimizer (GWO) [11], and Whale Optimization Algorithm (WOA)) [12]. Extensive experiments have been undertaken to contrast these two categories, focusing exclusively on HS, HLO, PRO, BA, GWO, and WOA, while testing them on various datasets.

Feature selection has seen a plethora of metaheuristic techniques tailored to address its challenges. Yusup et al. [13] compiled a good list of the HS method applications that have been used for feature selection problems. This includes solving a text clustering problem reported by Abualigah et al. [14], a Bangla word recognition problem reported by Das et al. [15] and non-technical loss detection reported by Ramos et al. [16]. The application of conventional HLO on feature selection is rare in the literature but its variants and extension to other algorithms have been reported. For example, a modified adaptive Human Learning Optimization Algorithm [17] and Differential Human Learning Optimization Algorithm [18] were reported in the literature. Similarly, the conventional PRO [9] algorithm has also limited applications to feature selection problems but its variant called hybrid Poor and Rich Optimization (HPRO) was reported by Thirumoorthy and Muneeswaran [19].

Mammal-based algorithms have been extensively applied for feature selection problems. The BA, for example, has been used for this application. However, its variants were also proposed for this application by Nakamura et al. with a new variant called the Binary Bat Algorithm [20]. Another application of BA was carried out on breast cancer datasets by Jeyasingh et al. [21]. Preti et al. [22] combined Discrete Cosine Transform (DCT)-Principal Component Analysis (PCA) with BA to solve a face recognition feature dimensionality problem. GWO feature selection with an SVM classifier was reported by Qasem et al. [23]. The algorithm was applied for feature selection on artillery disease. Applications of variants of GWO like Binary GWO on feature selection tasks are reported in Refs. [24,25]. Feature selection applications using WOA are reported [26,27]. Sharawi et al. [26] used a K-nearest neighbour (KNN) wrapper method to improve the feature classification quality. Zamani et al. [27] focused on feature selection for disease diagnosis. A binary variant of WOA was reported to solve the feature selection problem using wrapper feature selection by Majdi and Mirjalili [28].

Bacanin et al. [29] in their study addressed phishing website detection by introducing a diversity-oriented social network search algorithm within a two-level cooperative framework for feature selection and extreme learning model tuning. Their method outperformed six other metaheuristics, showcasing better average results for both challenges across multiple data sets. Sun et al. [30] proposed a hybrid metaheuristic feature selection technique named MetaSCA, based on the sine cosine algorithm (SCA) with added adjustments. The technique exhibited superior accuracy and optimal feature subset selection when tested on UCI data sets compared to three other algorithms. Kareem et al. [31] presented an enhanced feature selection method, GTO-BSA, combining Gorilla Troops Optimizer (GTO) with the bird swarm algorithm (BSA). Applied on IoT intrusion detection datasets, the GTO-BSA showed faster convergence and superior solutions compared to other contemporary techniques. Zivkovic et al. [32] introduced a swarm intelligence-based method for feature selection, utilizing a modified salp swarm algorithm. With a focus on K-nearest neighbors, the approach demonstrated improved feature selection and classification accuracy when tested against 21 datasets. Gharehchopogh et al. [33] enhanced the Vortex Search Algorithm (VSA) using chaos theory, specifically chaotic maps, to address its limitations. Applied on UCI datasets and as a feature selection tool, the modified VSA showed significant performance improvements and higher accuracy in real-world applications.

The core objective of this paper revolves around elucidating the capabilities of human behaviour and mammal-inspired optimization techniques in effectively addressing feature selection problems. The comparative analysis of these methods is undertaken thoroughly, with an emphasis on their explorative and computational prowess in the determination of optimal solutions. The major contributions of this paper are as follows.

• Application of six powerful and popular human behaviour-inspired and mammal-inspired optimization for the problem of feature selection.
• Application of diverse classification datasets to assess the applicability of these meta-heuristics on the problem of feature selection.
• Exhaustive comparative analysis of the selected metaheuristic algorithms by using feature selection as a benchmark problem.

The rest of the paper is organized as stated next. The methodology is discussed in Section 2. Section 3 covers the various evaluation criteria used in the paper. Section 4 i.e., the results and discussion section cover the results and comparative analysis of all six algorithms. Finally, the concluding remarks are given in Section 5.

## 2 Methodology

### 2.1 Nature-inspired optimization algorithms

In this study, the choice of metaheuristics in the paper focuses on algorithms inspired by both human behaviour and mammalian traits. Specifically, HS, HLO, PRO, BA, GWO and WOA are selected in this study for comprehensive comparison. The reason for selection of these algorithms is detailed below—

HS has a strong track record in optimization problems due to its unique exploration and exploitation mechanisms. It's known to find optimal solutions by considering a variety of possible solutions, akin to how musicians test different notes to create a harmonious piece.

HLO's adaptive learning strategies make it a compelling choice for complex problems where learning from past experiences can greatly guide the search process. Its human-centric approach can often resonate better with human-designed problems.

The dynamics of poor and rich can lead to a balance between exploration (searching new solutions) and exploitation (refining current solutions). Such dynamics can be quite effective in navigating complex search spaces.

The echolocation mechanism allows the algorithm to adjust its search space based on the quality of solutions, making it quite effective for problems where the solution space is vast and complex.

GWO leverages a combination of tracking, encircling, and attacking mechanisms inspired by wolf hunting behaviors. This combination can be highly effective in both converging to a solution and avoiding local optima.

The WOA's unique search mechanism allows for a balanced search that can adapt based on the proximity to the solution. This adaptability makes it a suitable choice for problems with complex landscapes.

#### 2.1.1 HS algorithm

HS algorithm is based on the spontaneous rhythm of music courses [7]. Musicians orchestrate their instruments to achieve the best score. Similar to any other metaheuristic technique in HS also, the vectors or parameters are tuned or updated over several iterations to find the best solution [34]. The implementation procedure for the HS algorithm can be summarized in these five simple steps.

STEP 1 Initialize harmony memory using equation (1)

(1) $$HM=\left[\begin{array}{ccc} x_{1}^{1},x_{2}^{1} & \cdots & x_{n}^{1}\\ \vdots & \ddots & \vdots \\ x_{1}^{HMS},x_{2}^{HMS} & \cdots & x_{n}^{HMS} \end{array}\right]$$

The matrix initializes a dimension of n size harmony which is referred to as HM.

STEP 2 Invent new harmony

New harmony is invented or improvised by memory consideration called Harmony Memory Consideration Rate (HMCR) and the pitching adjustment, pitching adjust rate.

STEP 3 The new harmony is included or disregarded
STEP 4 Repeat Step 2 and Step 3 until the final iteration
STEP 5 Optimal solution

The basic pseudocode of the HS method is reported in Algorithm 1.

**Figure:**
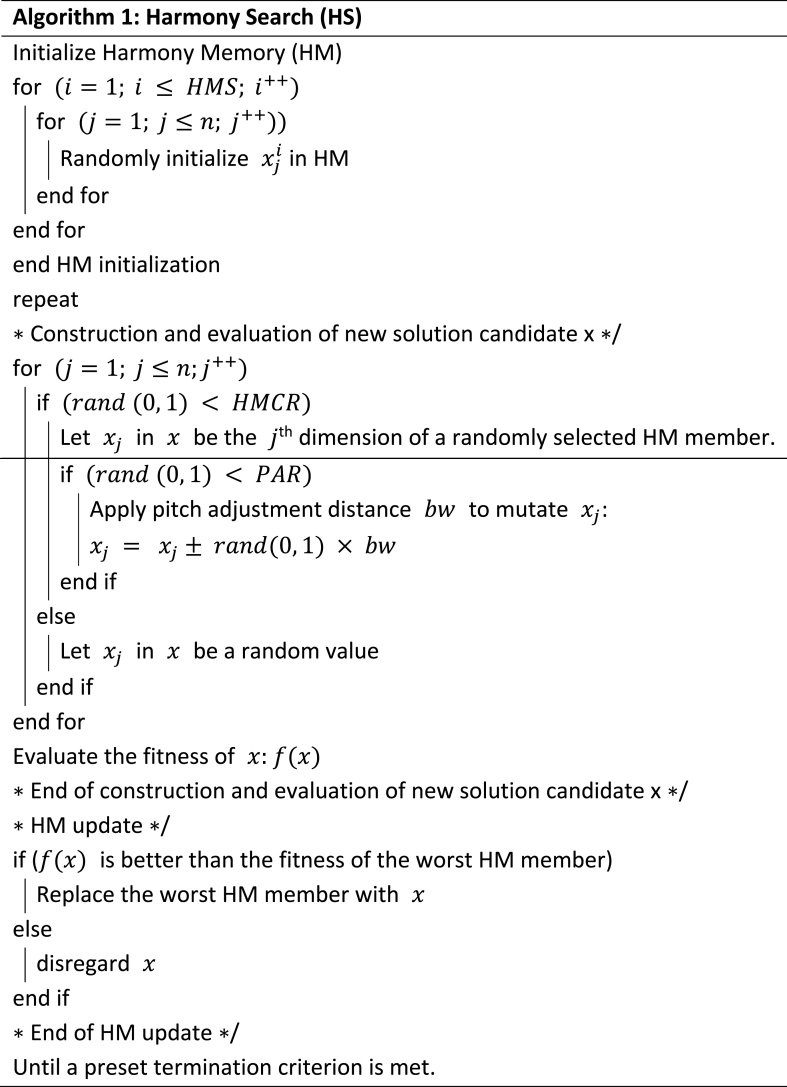

#### 2.1.2 HLO algorithm

The human learning process is extremely complicated. HLO, a metaheuristic inspired by this is a simplified version of the human learning process [8]. It considers four main learning factors, namely individual learning, social learning, random exploration learning and re-learning operative factors. Moreover, the algorithm is implemented in a binary form contrary to the continuous analogue of human behaviour. The reason is obvious since the learning optimization needs as simple procedures as possible in the metaheuristic optimization search in comparison to complex human learning skills of decision-making solutions.

The model starts with a binary string as shown in equation (2).

(2) $$x_{i}=[x_{i1}\ldots \ldots \text{,}x_{ij},\ldots ..x_{iM}\;with\;x_{ij}\in \left\{0,1\right\},1\leq i\leq N,1\leq j\leq M$$

Human random learning behaviour is implemented in the exploration phase using equation (3).

(3) $$x_{ij}=RE\left(0,1\right)=\left\{ \begin{array}{c} 0,r_{1}\leq 0.5\\ 1,else \end{array}\right.$$

Next step is the development of IndividualKnowledgeDatabase(IKD) which stores the best learning behaviour in equation (4).

(4) $${IKD}_{i}=\left[\begin{array}{c} {ikd}_{i1}\\ {ikd}_{i2}\\ \vdots \\ {ikd}_{ip}\\ \vdots \\ {ikd}_{iK} \end{array}\right]\left[\begin{array}{c} \begin{array}{ccc} {ik}_{i1,1} & \cdots & {ik}_{i1,j}\ldots {ik}_{i1,M}\\ \vdots & \ddots & \vdots \\ {ik}_{ip,1} & \cdots & {ik}_{ip,j}\ldots {ik}_{ip,M} \end{array}\\ \begin{array}{ccc} {ik}_{iK,1}\cdots & {ik}_{iK,j}\ldots & {ik}_{iK,M} \end{array} \end{array}\right]$$

When this is executed, equation (5) is applied.

(5) $$x_{ij}={ik}_{ip,j}$$

*Social Knowledge Database (SKD)* is utilized to restore the population's best understanding and is implemented through the use of equation (6).

(6) $${SKD}_{i}=\left[\begin{array}{c} {skd}_{1}\\ {skd}_{2}\\ \vdots \\ {skd}_{q}\\ \vdots \\ {skd}_{S} \end{array}\right]\left[\begin{array}{c} \begin{array}{ccc} {sk}_{11} & \cdots & {sk}_{1j}\ldots {sk}_{1M}\\ \vdots & \ddots & \vdots \\ {sk}_{q1} & \cdots & {sk}_{qj}\ldots {sk}_{qM} \end{array}\\ \begin{array}{ccc} {sk}_{S1}\cdots & {sk}_{Sj}\ldots & {sk}_{SM} \end{array} \end{array}\right]$$

To execute SKD, equation (7) is used.

(7) $$x_{ij}={sk}_{qj}$$

In summary, all are executed using equation (8) for a new solution.

(8) $$x_{ij}=\left\{ \begin{array}{c} RE\left(0,1\right),0\leq r\leq \mathrm{pr}\;\\ {ik}_{ip,j},\mathrm{pr}<r\leq pi\\ {sk}_{qj},else \end{array}\right.$$

The pseudocode of HLO method is reported in Algorithm 2.

**Figure:**
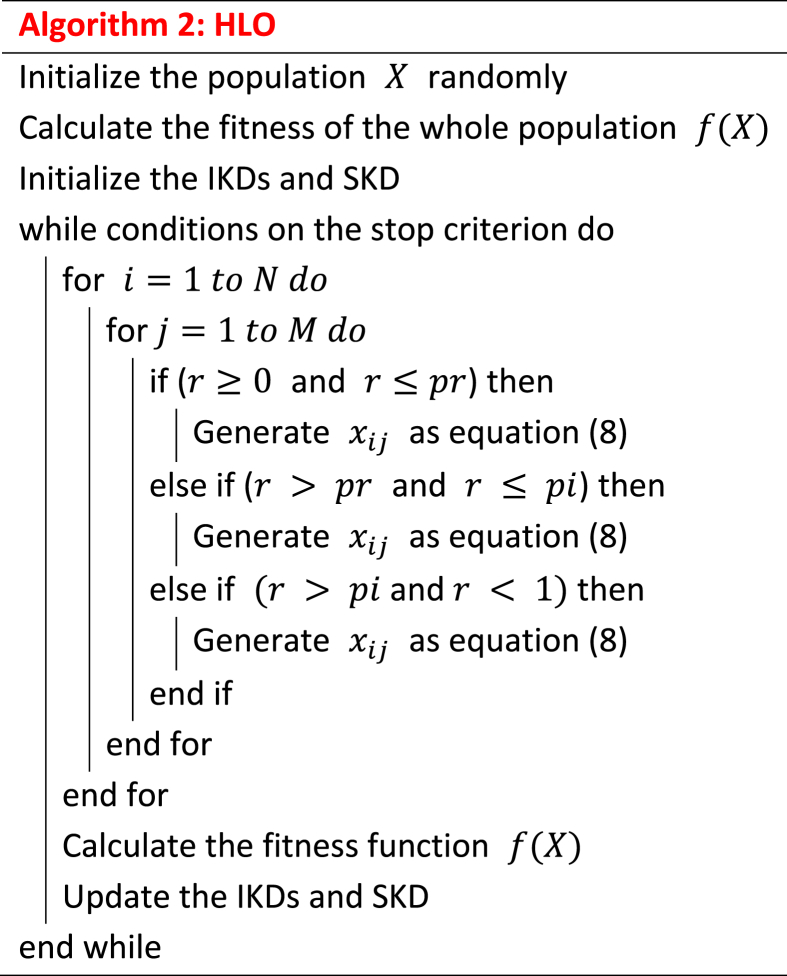

#### 2.1.3 PRO algorithm

PRO is based on human behaviour on affluence and abundance of material [9]. The rich strive to maintain or improve their level and the poor learn from the rich. Hence two kinds of common populations exist. These two populations are addressed as upper bound and lower bound in the PRO model. In general, the algorithm is implemented by using the following procedure.

STEP 1 The population is sorted into two categories-poor and rich, as shown in equation (9)

(9) $${POP}_{main}={POP}_{poors}+{POP}_{riches}$$

STEP 2 Change of position in each group as shown in equation (10)

(10) $$\vec{X_{rich,1}^{new}}=\vec{X_{rich,1}^{old}}+r\times \left[\vec{X_{rich,1}^{old}}-\vec{X_{poor,best}^{old}}\right]$$

The process of updating occurs in this step. While the rich population is updated according to equation (10), the poor is updated using equation (11).

(11) $$\vec{X_{poor,i}^{new}}=\vec{X_{poor,i}^{old}}+\left[r(Pattern]-X_{poor,i}^{old}\right]$$

The pattern is defined using the following equation (12)

(12) $$Pattern=\frac{\vec{X_{rich,best}^{old}}+\vec{X_{rich,mean}^{old}}+\vec{X_{rich,worst}^{old}}}{3}$$

STEP 3 Mutation

In this phase, the mutation step is performed based on the rise and fall prediction of the economy.

The step is modelled in equations (13), (14) for both populations.

(13) $$\left\{ \begin{array}{c} if\;rand<Pmut\\ \vec{X_{rich,i}^{new}}=\vec{X_{rich,i}^{new}}+randn\\ end \end{array}\right.$$

(14) $$\left\{ \begin{array}{c} if\;rand<Pmut\\ \vec{X_{poor,i}^{new}}=\vec{X_{poor,i}^{new}}+randn\\ end \end{array}\right.$$

STEP 4 Best Rich is achieved

The algorithm ends at this stage with a new population of poor and rich.

The pseudocode of the PRO method is described in Algorithm 3.

**Figure:**
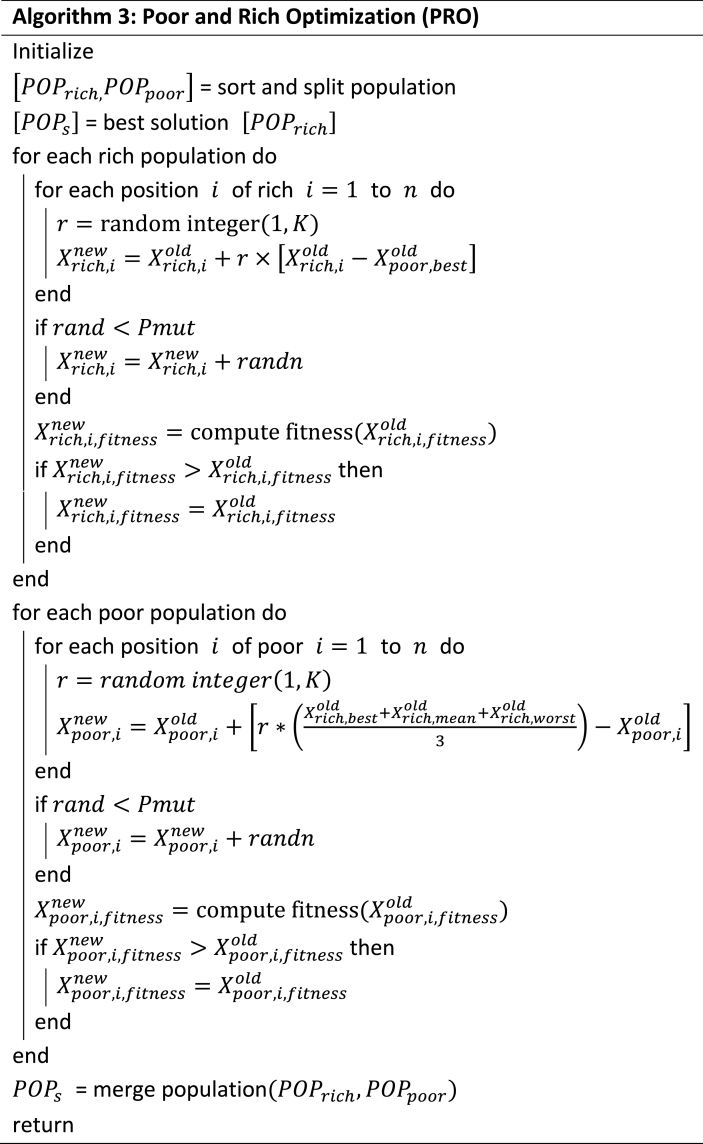

#### 2.1.4 Bat Algorithm

The algorithm is grounded in the natural possession of sonar technology by bats [10]. Sonar, also known as echolocation, provides bats with the capability to hunt in complete darkness. This distinct echolocation capability is the target in the Bat Algorithm to attain the objective function [35]. The model of this Bat Algorithm is formulated according to the following equations and the pseudocode for this algorithm is shown in Algorithm 4.

As with other metaheuristic algorithms, n arbitrary bats population is created and initialized in dimension d as shown in equation (15).

(15) $$x_{i,j}=x_{\min,j}+rand\left(0,1\right)\left(x_{maxj}-x_{minj}\right)$$

where i=1,2,….,n and j=1,…..,d. $x_{minj}$ are lower boundaries and $x_{maxj}$ are upper boundaries.

The updates of the process of frequency velocity and solution are as shown in equations (16), (17), (18) [36].

(16) $$f_{i}=f_{\min}+\left(f_{\max}-f_{\min}\right)\beta$$

(17) $$v_{i}^{t}=v_{i}^{t-1}+\left(x_{i}^{t}-x_{*}\right)f_{i}$$

(18) $$x_{i}^{t}=x_{i}^{t-1}+v_{i}^{t}$$

where βϵ[0,1] is a random number and $x_{*}$ are current global best solutions. For the exploitation phase, one solution is selected among the best solution and a random walk is applied. Equation (19) represents this step.

(19) $$x_{new}=x_{old}+\varepsilon A^{t}$$

where ε∈[0.1] is a random number that represents the direction and intensity of a random walk and $A^{t}$ is average of bats loudness. The loudness and pulse emission are then updated as represented in equations (20), (21).

(20) $$A_{i}^{t+1}=\alpha A_{i}^{t}$$

(21) $$r_{i}^{t+1}=r_{i}^{0}\left[1-e^{\left(-\gamma t\right)}\right]$$

where α, γ are constants and $A_{i}^{t}$ initially can range from Refs. [1,2] and $r_{i}^{0}\;\text{is}$ between [0,1].

**Figure:**
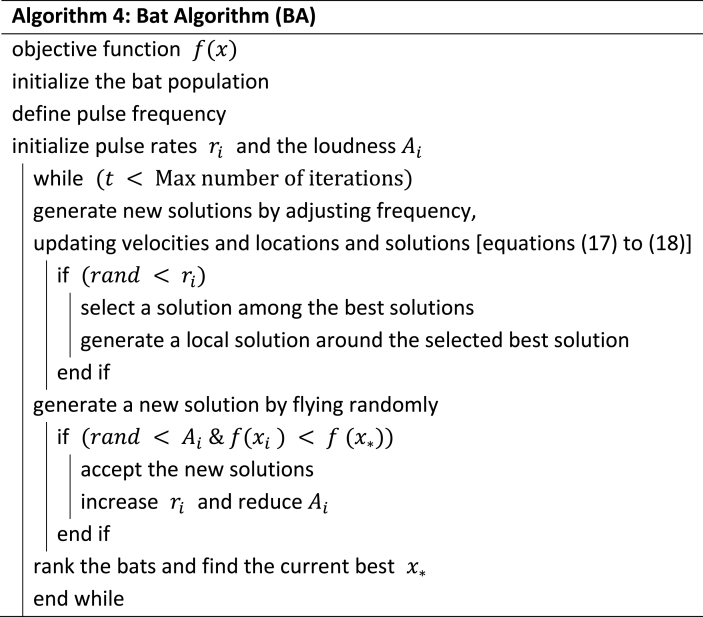

#### 2.1.5 GWO algorithm

This optimizer algorithm is based on the Grey Wolf group or pack which usually consists of five to twelves member of wolves. It has a unique association with the wolf's teamwork which each wolf can categorized as alpha, beta, delta and omega wolf [11].

This behaviour of the wolves is expressed in the following mathematical expressions and its pseudocode is presented in Algorithm 5. Details of this algorithm can be found in Ref. [37] and its original initiator in Ref. [11].

(22) $$\vec{A}=2\vec{a}∙\vec{r_{1}}-\vec{a}$$

(23) $$\vec{B}=2\vec{r_{2}}$$

(24) $$\vec{D_{\alpha }}=\left|\vec{B}∙\vec{X_{\alpha }}-\vec{X_{i}}\right|,\vec{D_{\beta }}=\left|\vec{B}∙\vec{X_{\beta }}-\vec{X_{i}}\right|,\vec{D_{\delta }}=\left|\vec{B}∙\vec{X_{\delta }}-\vec{X_{i}}\right|$$

(25) $$\vec{X_{1}}=\vec{X_{\alpha }}-\vec{A}∙\vec{D_{\alpha }},\vec{X_{2}}=\vec{X_{\beta }}-\vec{A}∙\vec{D_{\beta }},\vec{X_{3}}=\vec{X_{\delta }}-\vec{A}∙\vec{D_{\delta }}$$

Equations (22)–(25) are mathematical models that express the three alpha, beta and delta wolves. They are updated using equation (26).

(26) $$\vec{X_{i}}\left(nt\right)=\frac{\vec{X_{1}}+\vec{X_{2}}+\vec{X_{3}}}{3}$$

a is then updated using equation (27)

(27) a=2−2*it/MAX_IT

**Figure:**
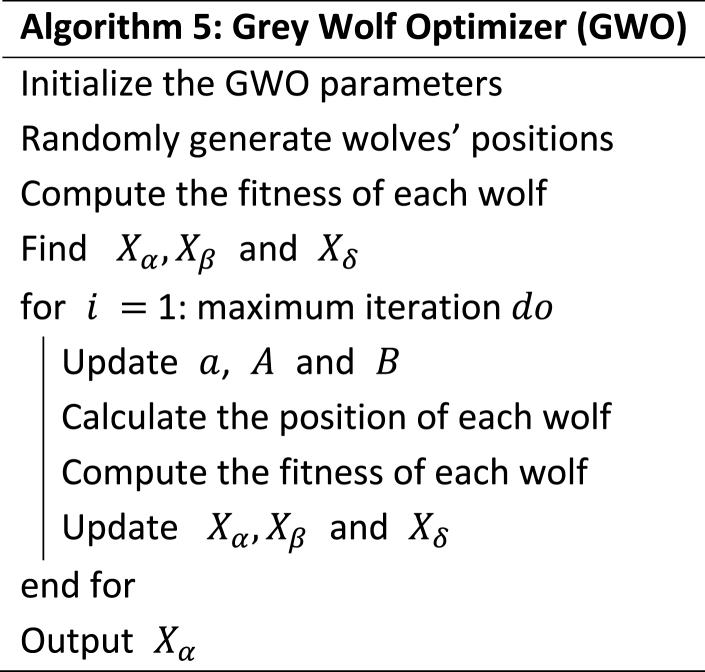

#### 2.1.6 Whale Optimization Algorithm

As the name implies, the humpbackwhales preying social behaviour is the background of the WOA method [12]. The preying behaviour is simple, it can be summarized into only two steps-encirclement and searching for the prey [38].

The whales when in the condition of preying is modelled by equations (28), (29).

(28) $$\vec{D}=\left|\vec{C}.\vec{X^{*}\left(t\right)-X\left(t\right)}\right|$$

in which t denotes the usual iterations, X is the current solution and $X^{*}$ is the best value.

(29) $$\vec{X}\left(t+1\right)=\vec{X^{*}}\left(t\right)-\vec{A}∙\vec{D}$$

$\vec{A}$ and $\vec{C}$ are normal algebraic constant expressions which are expressed in equations (30), (31).

(30) $$\vec{A}=2\vec{a}∙\vec{r}-\vec{a}$$

(31) $$\vec{C}=2\vec{r}$$

Encircled behaviour is then shrunk of $\vec{a}$ value from equation (30) through the expression of equation (31) which resulted in equation (32).

(32) $$\vec{a}=2-t\frac{2}{MaxIter}$$

These calculations, thus achieve the exploitation phase by measuring the distance of X, the current location and the best solution, $X^{*}$. Through this measurement, a spiral equation is created as modelled in equation (33).

(33) $$\vec{X}\left(t+1\right)=D^{\prime }∙e^{bl}∙\cos\left(2\pi l\right)+\vec{X^{*}}\left(t\right)$$

These mechanisms are expressed in equation (34) with a 50 % probability assumed.

(34) $$\vec{X}\left(t+1\right)=\left\{ \begin{array}{c} shrinking\;encircling\;\left(equation\;\left(2\right)\right)\;if\;\left(p<0.5\right)\\ spiral-shaped\;path\;\left(equation\left(6\right)\right)\;if\;\left(p\geq 0.5\right) \end{array}\right.$$

The position is then updated by equations (35), (36).

(35) $$\vec{D}=\left|\vec{C}∙\vec{X_{rand}}-\vec{X}\right|$$

(36) $$\vec{X}\left(t+1\right)=\vec{X_{\mathrm{ran}d}}-\vec{A}∙\vec{D}$$

The pseudocode of this algorithm is described in Algorithm 6.

**Figure:**
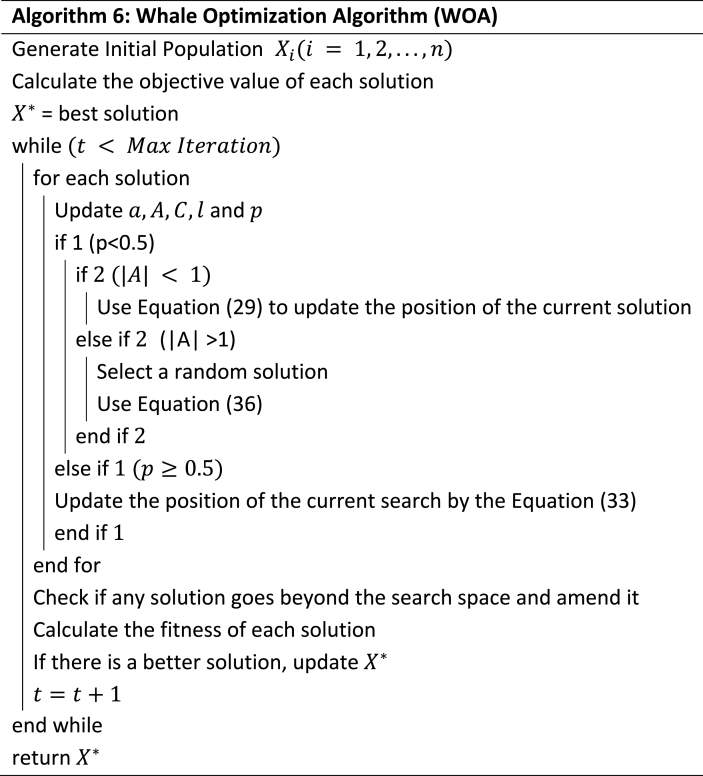

## 3 Evaluation criteria

To evaluate the algorithm performance on the datasets the following measurements were applied.

### 3.1 Classification accuracy

Average accuracy is measured to the model accuracy calculation when finding the correct category of feature subsets. Equation (37) for estimating average classification accuracy is given as follows:

(37) $$\text{Average}\;\text{Accuracy}=\frac{1}{M}\sum_{j=1}^{M}\frac{1}{N}\sum_{i=1}^{N}match(C_{i},L_{i})$$

where M is the number of runs, N is the test setpoint number. The word match means comparator which returns 0 when two labels are not identical and returns 1 when identical. $C_{i}$ is data point output label and $L_{i}$ is reference label for i.

### 3.2 Average selected features

Average selected features refer to n features averaged over M times and is calculated as per equation (38)

(38) $$\text{Average}\;\text{Selected}\;\text{Features}=\frac{1}{M}\sum_{i=1}^{M}size\left(g^{*}\right)$$

where $size\left(g^{*}\right)$ is the number of testing datasets attributes.

### 3.3 Statistical Mean

Statistical Mean is the average obtained when running each method over *M* times as shown in equation (39)

(39) $$\text{Mean}=\frac{1}{M}\sum_{i=1}^{M}size\left(g^{*i}\right)$$

where $g^{*i}$ = optimal fitness over $i^{th\;}run$.

### 3.4 Statistical standard deviation

It is the standard deviation dispersed over the mean which can be expressed as follow equation (40)

(40) $$\text{STD}=\sqrt{\frac{1}{M-1}\sum_{i=1}^{M}{\left(size\left(g^{*i}\right)-Mean\right)}^{2}}$$

### 3.5 Average run time

Algorithm real running time is expressed as the following equation (41)

(41) $$\text{Average}\;\text{RT}=\frac{1}{M}\sum_{i=1}^{M}{RT}_{o,i}$$

## 4 Results and discussion

### 4.1 Parameter description

These six metaheuristic techniques were run and executed on various datasets to evaluate their performances. The datasets chosen are tabulated in Table 1. Parameters for the testing are restricted and common for all the six algorithms as described in Table 2. However, additional parameters for the HS algorithm like pitching adjust rate, harmony memory considering rate and bandwidth are assigned values as in Table 2. The same goes for special attributes of HLO, PRO, BA, GWO and WOA which were initially assigned before the experiment. The common parameters assigned for all algorithms are— k = 5 which refers to the number of nearest neighbors considered. The value 5 is commonly used in literature as it often provides a balance between underfitting and overfitting. Next, the maximum iterations of 200 was chosen to ensure convergence of the algorithms without incurring excessive computational cost. The number of search agents was chosen as 30 to maintain diversity and avoid premature convergence. The number of independent runs is 20 i.e., each experiment is repeated 20 times with new seed values. Multiple runs provide a more accurate estimate of the algorithms' performance, and 20 is a standard number of runs in the literature. The ratio of validation data is kept as 20 % to ensure sufficient data for validation without compromising the training set size.

**Table 1:** Details of the datasets used in this work.

Dataset	Number of Instances	Number of features
Heart	303	13
Sonar	206	60
German	1000	24
Ionosphere	351	34
Breast Cancer	699	9
Ovarian Cancer	216	4000

**Table 2:** Parameter settings of all algorithms.

Algorithm	Parameter	Value
Common for all algorithms	k	5
	Maximum Iterations	200
	Number of Search Agents	30
	Number of Independent Runs	20
	Ratio of Validation Data	0.2
Harmony Search (HS)	Pitch Adjusting Rate	0.05
	Harmony Memory Considering Rate	0.7
	Bandwidth	0.2
Human Learning Optimization (HLO)	Probability of Individual Learning	0.85
	Probability of Exploration Learning	0.1
Poor And Rich Optimization (PRO)	Mutation Probability	0.06
Bat Algorithm (BA)	Maximum Frequency	2
	Minimum Frequency	0
	α	0.9
	γ	0.9
	Maximum Loudness	2
	Maximum Pulse Rate	1
Whale Optimization Algorithm (WOA)	b	1

Apart from the common parameters, specific parameters were set for all the six algorithms. The selected parameters for HS are based on the original work introducing the algorithm and subsequent empirical studies that found these values to result in optimal performance. The probability of individual learning of 0.85 and probability of exploration learning of 0.1 are set according to foundational papers on HLO. These values ensure a balance between exploration and exploitation in the search space. In case of PRO, the mutation probability of 0.06 was determined empirically to yield optimal results in our initial tests. The parameters for BA, including the frequency and loudness values, are in line with the original proposal of the BA. These settings ensure that bats can explore the search space effectively. The parameter for WOA was chosen in line with its original research paper, which found this value to be effective across various optimization problems.

### 4.2 Fitness analysis

**Fig. 1:**
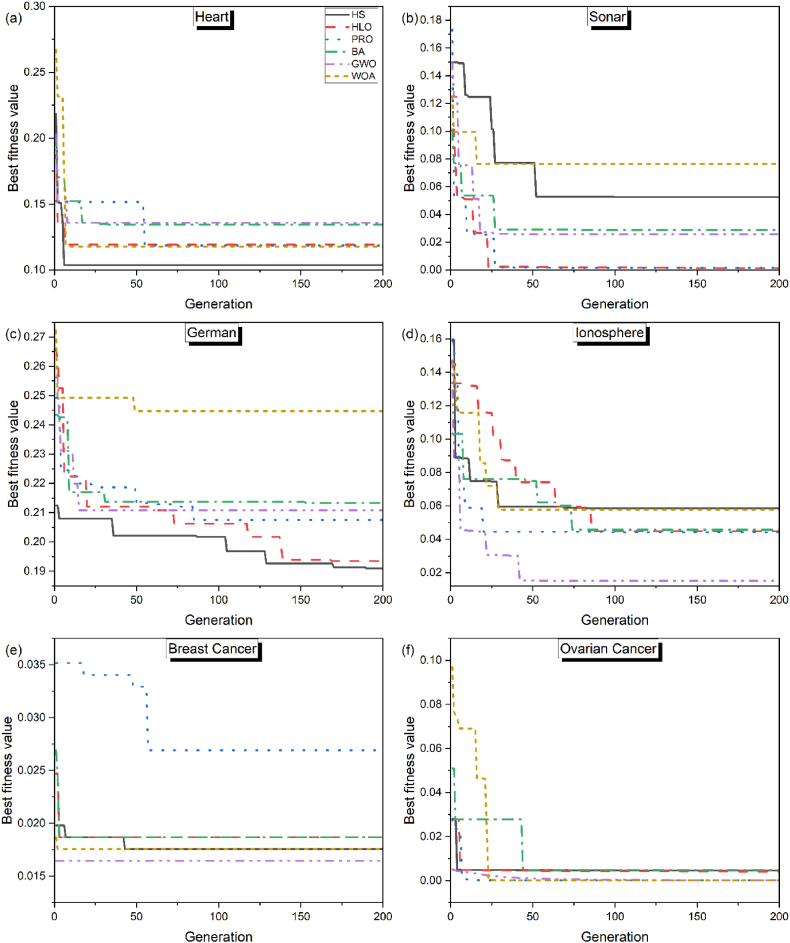
Convergence curves of all algorithms for (a) Heart dataset, (b) Sonar dataset, (c) German dataset, (d) Ionosphere dataset, (e) Breast Cancer dataset, (f) Ovarian Cancer dataset.

The results of the experiment are shown in Table 3 which is the best fitness values of all algorithms for all datasets. The average (of all datasets) rank and median (of all datasets) rank of the algorithms is also reported for ease of comprehension. HLO and HS performed better in comparison to other algorithms. The convergence curves are illustrated in Fig. 1(a–f), displaying the overall results for each dataset by all algorithms. In terms of the best fitness, the algorithms can be ranked as HLO > HS > PRO > WOA > GWO > BA. However, it is worth mentioning here that on the Ovarian Cancer dataset which contains a very large number of features, the WOA and GWO marginally outperform PRO and comprehensibly outperform HLO and HS.

**Table 3:** Best fitness values of all algorithms for all datasets.

Dataset	HS	HLO	PRO	BA	GWO	WOA
Heart	**0.0376**	0.0518	0.1029	0.1021	0.1178	0.0856
Sonar	0.0028	0.0013	**0.0008**	0.0278	0.0015	0.0283
German	**0.1774**	0.1935	0.1919	0.2038	0.1964	0.2092
Ionosphere	0.0445	**0.0021**	0.0147	0.0457	0.0150	0.0292
Breast Cancer	**0.0056**	0.0105	0.0116	0.0105	0.0165	0.0105
Ovarian Cancer	0.0045	0.0042	0.0002	0.0047	0.0001	**0.0000**
Mean Rank	2.8	**2.5**	3.0	4.8	4.0	3.8
Median Rank	**2.5**	**2.5**	**2.5**	5.0	3.5	3.5

Descriptive statistics analysis of mean and standard deviation of fitness values for each algorithm for all datasets are elaborated in Table 4 and Table 5 respectively. From Tables 4 and it is clear that the human behaviour-inspired algorithms have better performance as compared to the mammal-inspired algorithms. However, on a large feature selection dataset (Ovarian Cancer) the performance of PRO is at par with GWO and marginally better than WOA. Nevertheless, based on the six datasets considered in this study the algorithms can be ranked on mean fitness values as HLO > PRO > HS > GWO > WOA > BA. In terms of the deviation of fitness values upon multiple trials, the results show that HLO > GWO > PRO > BA > WOA > HS.

**Table 4:** Mean fitness values of all algorithms for all datasets.

Dataset	HS	HLO	PRO	BA	GWO	WOA
Heart	0.1058	**0.0925**	0.1192	0.1354	0.1390	0.1390
Sonar	0.0321	**0.0018**	0.0210	0.0377	0.0210	0.0569
German	**0.1952**	0.2028	0.2124	0.2180	0.2110	0.2376
Ionosphere	0.0619	**0.0362**	0.0379	0.0658	0.0383	0.0577
Breast Cancer	0.0161	**0.0156**	0.0291	0.0244	0.0249	0.0213
Ovarian Cancer	0.0184	0.0089	**0.0002**	0.0140	**0.0002**	0.0003
Mean Rank	3.3	1.7	3.1	4.8	3.6	4.5
Median Rank	3.0	1.0	2.8	5.0	3.0	4.5

**Table 5:** STD of fitness values of all algorithms for all datasets.

Dataset	HS	HLO	PRO	BA	GWO	WOA
Heart	0.0434	0.0306	**0.0112**	0.0328	0.0188	0.0462
Sonar	0.0206	**0.0003**	0.0203	0.0129	0.0204	0.0197
German	0.0117	**0.0067**	0.0194	0.0138	0.0162	0.0193
Ionosphere	**0.0159**	0.0274	0.0161	0.0166	0.0164	0.0224
Breast Cancer	0.0086	**0.0060**	0.0150	0.0119	0.0083	0.0083
Ovarian Cancer	0.0127	0.0104	**0.0000**	0.0126	0.0000	0.0002
Mean Rank	4.0	2.7	3.3	3.8	3.0	4.2
Median Rank	4.5	2.0	3.0	4.0	2.5	4.0

Table 6 shows the worst fitness among the best fitness for 20 independent trials for each algorithm on each dataset. The inclusion of the worst values alongside the best, mean and standard deviation can provide a comprehensive picture of the algorithm's behaviour. This data point would highlight the lower bound of the algorithm's performance, helping researchers and practitioners gauge the reliability and consistency of an algorithm. It is observed from Table 6 that HLO > PRO > GWO > HS > WOA > BA.

**Table 6:** Worst fitness values of all algorithms for all datasets.

Dataset	HS	HLO	PRO	BA	GWO	WOA
Heart	0.1516	**0.1194**	0.1343	0.1689	0.1696	0.2026
Sonar	0.0525	**0.0022**	0.0493	0.0520	0.0503	0.0764
German	**0.2075**	0.2125	0.2343	0.2409	0.2360	0.2624
Ionosphere	0.0881	0.0728	**0.0572**	0.0890	0.0583	0.0863
Breast Cancer	0.0269	**0.0247**	0.0532	0.0401	0.0340	0.0329
Ovarian Cancer	0.0278	0.0274	**0.0002**	0.0278	0.0002	0.0006
Mean Rank	3.6	2.0	2.6	4.9	3.3	4.7
Median Rank	4.0	1.5	2.0	5.0	3.5	5.0

### 4.3 Classification accuracy analysis

The average classification accuracy of all algorithms for all datasets is represented in Table 7 and its standard deviation is described in Table 8. HLO again outperform the rest in the Heart, Sonar, Ionosphere and Breast Cancer datasets as shown in Table 7, whereas on the German dataset HS performs the best. Interestingly, in the case of a large number of feature datasets i.e., the Ovarian Cancer dataset all the algorithms perform very well with near ideal accuracy for HS, HLO and BA whereas the ideal accuracy of 100 % classification rate for PRO, GWO and WOA. Moreover, the standard deviation of this dataset is also zero for PRO, GWO and WOA indicating that these algorithms achieved 100 % classification in all 20 independent trails. Thus, based on average classification accuracy, the algorithms are ranked as HLO > PRO ∼ GWO > HS > WOA > BA and based on STD classification accuracy as HLO > GWO > HS > BA > PRO > WOA.

**Table 7:** Average classification accuracy of all algorithms for all datasets.

Dataset	HS	HLO	PRO	BA	GWO	WOA
Heart	0.897	**0.910**	0.883	0.867	0.863	0.863
Sonar	0.971	**1.000**	0.980	0.966	0.980	0.946
German	**0.807**	0.800	0.789	0.784	0.790	0.766
Ionosphere	0.940	**0.966**	0.963	0.937	0.963	0.943
Breast Cancer	0.988	**0.988**	0.976	0.980	0.978	0.983
Ovarian Cancer	0.986	0.995	**1.000**	0.991	**1.000**	**1.000**
Mean Rank	3.3	1.7	3.3	4.8	3.3	4.5
Median Rank	3.0	1.0	2.8	5.0	2.8	5.0

**Table 8:** STD classification accuracy of all algorithms for all datasets.

Dataset	HS	HLO	PRO	BA	GWO	WOA
Heart	0.045	0.030	**0.012**	0.033	0.018	0.046
Sonar	0.020	**0.000**	0.020	0.013	0.020	0.020
German	0.012	**0.007**	0.020	0.015	0.017	0.020
Ionosphere	**0.016**	0.028	0.016	0.016	0.016	0.023
Breast Cancer	0.008	**0.006**	0.016	0.012	0.007	0.008
Ovarian Cancer	0.013	0.010	**0.000**	0.013	**0.000**	**0.000**
Mean Rank	3.4	4.3	3.2	3.3	4.0	2.8
Median Rank	3.0	5.0	3.0	3.5	4.5	2.5

**Fig. 2:**
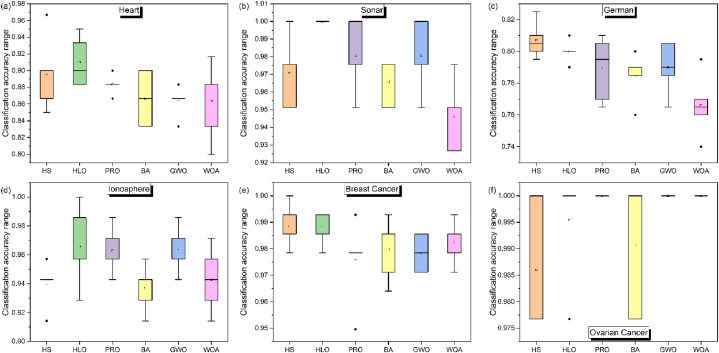
Boxplot analysis (20 independent trials) of classification accuracy of all algorithms for (a) Heart dataset, (b) Sonar dataset, (c) German dataset, (d) Ionosphere dataset, (e) Breast Cancer dataset, (f) Ovarian Cancer dataset.

A further analysis was made on the metaheuristic techniques classification accuracy. Fig. 2(a–f) showed the Boxplot analysis of the classification accuracy of all algorithms for all datasets. It is observed that in general, BA has the most deviation in repeated trials. PRO is observed to be most robust to repeated trials followed by GWO.

### 4.4 Analysis of feature reduction

Experimental results of the selected feature subsets with statistical analysis of all algorithms for all datasets in regards to their mean and standard deviation are illustrated in Table 9 and Table 10 respectively. The percentage reduction in features with respect to the original feature dimensions is reported within the brackets in Table 9. It is observed that for datasets with a very low (<15) number of features (like the Heart dataset and Breast Cancer dataset), the performance of all the algorithms is very similar. For example, the difference between the best value (HLO/BA) versus the worst value (PRO/GWO/WOA) for the Heart dataset is only 3.1 %. For the Sonar dataset which has 60 features, a significant deviation in the performance of algorithms is seen. Around 22 % difference between the best (PRO/GWO) and worst (BA) performance is observed for the Sonar dataset. On a very large feature dataset i.e., the Ovarian Cancer dataset which has 4000 features, the difference between the best (PRO) and worst (BA) performance is observed to be 46 %. Overall, the performance of PRO and GWO is found to be best in reducing the number of features whereas BA is reported to have performed the worst feature reduction among the tested algorithms. Thus, considering both the % reduction in features and the standard deviation of reduction, the algorithms can be ranked as PRO > GWO > WOA > HLO > HS > BA.

**Table 9:** Mean selected feature subsets (and % reduction in features) of all algorithms for all datasets.

Dataset	HS	HLO	PRO	BA	GWO	WOA
Heart	4.6 (64.6 %)	4.4 (66.2 %)	4.8 (63.1 %)	4.4 (66.2 %)	4.8 (63.1 %)	4.8 (63.1 %)
Sonar	18.6 (69 %)	10.6 (82.3 %)	10.2 (83 %)	23.6 (60.7 %)	10.2 (83 %)	22.8 (62 %)
German	9.8 (59.2 %)	11.4 (52.5 %)	8.4 (65 %)	10 (58.3 %)	7.4 (69.2 %)	14.2 (40.8 %)
Ionosphere	8.4 (75.3 %)	7.8 (77.1 %)	3.8 (88.8 %)	12 (64.7 %)	5.2 (84.7 %)	4 (88.2 %)
Breast Cancer	4.2 (53.3 %)	3.8 (57.8 %)	4.4 (51.1 %)	4 (55.6 %)	3.2 (64.4 %)	3.8 (57.8 %)
Ovarian Cancer	1832.8 (54.2 %)	1711.6 (57.2 %)	64 (98.4 %)	1905 (52.4 %)	64.8 (98.4 %)	112.4 (97.2 %)
Average reduction	62.6 %	65.5 %	74.9 %	59.6 %	77.1 %	68.2 %

**Table 10:** STD of the selected feature subsets of all algorithms for all datasets.

Dataset	HS	HLO	PRO	BA	GWO	WOA
Heart	1.49	0.83	1.23	0.84	1.09	1.31
Sonar	1.01	1.13	5.03	1.58	1.09	4.93
German	0.59	0.73	2.30	2.97	1.50	1.53
Ionosphere	1.79	1.92	1.66	2.05	1.12	1.58
Breast Cancer	1.51	0.83	1.09	0.71	1.13	1.23
Ovarian Cancer	44.50	37.44	2.70	9.88	11.03	79.61

### 4.5 Run time analysis

**Fig. 3:**
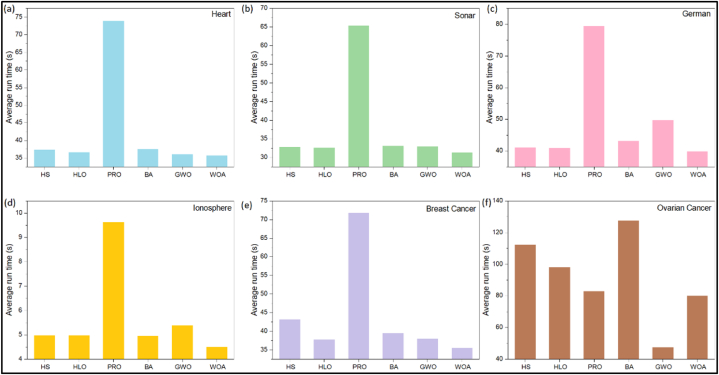
Average run time of all algorithms for (a) Heart dataset, (b) Sonar dataset, (c) German dataset, (d) Ionosphere dataset, (e) Breast Cancer dataset, (f) Ovarian Cancer dataset.

Fig. 3(a–f) displayed the average run time of all algorithms for all datasets. PRO took a longer run time for the datasets with BA took a longer time in the Ovarian Cancer dataset. As anticipated, due to a higher number of features, all the algorithms took a longer time in the Ovarian Cancer dataset. But, GWO was observed to be remarkably faster than others in this large-scale feature dataset as well. In terms of computational time, overall, the algorithms can be ranked as WOA > GWO > HLO > HS > BA > PRO.

### 4.6 Comparison with recent state of the art

Table 11 presents a comparison of the average feature reduction percentages achieved by various algorithms across multiple datasets. The PRO and GWO algorithms appear to outperform most of the other algorithms, particularly when observing the results on the Ovarian cancer dataset, where they both achieve feature reductions of 98 %. It indicates that these algorithms work well especially for features with large number of features. On the other hand, HLO seems to have a consistent performance across various datasets. It offers an above-average reduction percentage in most cases, with its highest feature reduction being 82 % on the Sonar dataset. Comparing the current algorithms with the ones referenced from Refs. [39,40], it is evident that the current algorithms (like PRO, GWO, etc.) typically outperform or are at least on par with the older ones. For instance, on the Ionosphere dataset, PRO achieves an impressive 89 % feature reduction, surpassing the results of GA, DE, and PSO from Ref. [40].

**Table 11:** Comparison of average feature reduction % by various algorithms.

Method	Breast cancer	Ionosphere	Sonar	Heart	German	Ovarian cancer
HS	53 %	75 %	69 %	65 %	59 %	54 %
HLO	58 %	77 %	82 %	66 %	53 %	57 %
PRO	51 %	**89 %**	**83 %**	63 %	65 %	**98 %**
BA	56 %	65 %	61 %	66 %	58 %	52 %
GWO	**64 %**	85 %	**83 %**	63 %	**69 %**	98 %
WOA	58 %	88 %	62 %	63 %	41 %	97 %
SA [39]	40 %	66 %	57 %	66 %	60 %	50 %
GSA [39]	60 %	71 %	60 %	66 %	58 %	53 %
ASO [39]	53 %	69 %	70 %	63 %	51 %	58 %
DE [40]	51 %	55 %	45 %	66 %	39 %	39 %
GA [40]	60 %	71 %	75 %	**69 %**	51 %	66 %
PSO [40]	44 %	69 %	72 %	65 %	47 %	54 %
FPA [40]	51 %	58 %	64 %	62 %	54 %	52 %
MRFO [40]	62 %	85 %	73 %	65 %	53 %	98 %
WSA [40]	51 %	48 %	48 %	**69 %**	39 %	50 %

### 4.7 Discussions

Metaheuristic techniques have emerged as potent tools for wrap-based dimensionality reduction, offering the capability to efficiently navigate the vast search space of data. With their guided exploration, they can pinpoint optimized solutions, especially for intricate problems such as feature selection.

In this study, we delved deep into the comparative performance of human-behaviour-based and mammal-based metaheuristic techniques concerning feature selection. This comparative approach revealed multifaceted insights, illustrating the idiosyncrasies of each technique when applied to a diverse array of datasets.

It is crucial to note that seeking an overarching, universal solution encompassing all evaluative metrics—like accuracy, feature reduction percentage, and computational efficiency—isn't realistic due to the inherent complexities and unique characteristics of each technique. For instance, while one method might prioritize accuracy, another may be optimized for speed.

However, amidst this diversity in performance, some techniques, notably PRO and HLO, exhibited commendable adaptability, registering notable achievements across several evaluation criteria.

A summary of the standout techniques across various performance metrics is presented as follows.

• Best Fitness Score: HLO emerged as the top contender, closely followed by HS and then PRO.
• Mean Fitness Score: HLO took the lead once again, with PRO and HS coming in subsequent positions.
• Deviation in Fitness on Repeated Trials: HLO proved its consistent performance, leading the pack, with GWO and PRO taking the subsequent spots.
• Worst Fitness Score: The ranking, in this case, was spearheaded by HLO, trailed by PRO and GWO.
• Average Classification Accuracy: HLO remained at the forefront, with PRO and GWO almost neck-to-neck in their performances.
• Deviation in Classification Accuracy on Repeated Trials: HLO emerged as the most consistent, followed by GWO, and then HS.
• Feature Reduction Efficiency: PRO was the clear leader, with GWO and WOA filling the next two spots.
• Computational Time Efficiency: WOA outperformed others in speed, followed by GWO, and then HLO.

## 5 Conclusions

In this research, various metaheuristic techniques inspired by human behaviour and mammalian traits were compared for their efficiency in feature selection problems. It became evident that while there isn't a one-size-fits-all solution, certain techniques like PRO and HLO consistently showcased promising results across various metrics. Notably, human-inspired metaheuristics like PRO demonstrated exceptional potential in dimensionality reduction without compromising classification accuracy, and often outperformed established algorithms like GWO. While the study delves into a select few metaheuristics, it opens avenues for exploration of hybrid variants. The challenge remains for researchers to design a universal algorithm excelling in all dimensions.

In line with the 'No-Free-Lunch' theory, our study reinforces the understanding that no single algorithm is universally superior. The performance of an algorithm greatly depends on the problem specifics and dataset characteristics. Thus, while certain algorithms may shine in specific scenarios or datasets, it's imperative for practitioners to consider the unique nature of their problem when choosing a metaheuristic for feature selection.

## Funding

This research received no external funding.

## Institutional review board statement

Not applicable.

## Informed consent statement

Not applicable.

## Data availability statement

The data presented in this study are available through email upon request to the corresponding author.

## Additional information

No additional information is available for this paper.

21 of 22.

## CRediT authorship contribution statement

**Mariappan Premalatha:** Conceptualization, Data curation, Formal analysis, Investigation, Validation, Writing – original draft, Writing – review & editing. **Murugan Jayasudha:** Formal analysis, Investigation, Methodology. **Robert Čep:** Investigation, Methodology, Supervision, Writing – original draft, Writing – review & editing. **Jayaraju Priyadarshini:** Investigation, Methodology, Writing – original draft. **Kanak Kalita:** Conceptualization, Data curation, Investigation, Writing – original draft, Writing – review & editing. **Prasenjit Chatterjee:** Conceptualization, Formal analysis, Investigation, Methodology, Supervision, Validation, Writing – original draft, Writing – review & editing.

## Declaration of competing interest

The authors declare that they have no known competing financial interests or personal relationships that could have appeared to influence the work reported in this paper.

## References

[1] Dhal P., Azad C. (2022).

[2] Dokeroglu T., Deniz A., Kiziloz H.E. (2022).

[3] Gunantara N., Nyoman D., Putra N. (2019). The characteristics of metaheuristic method in selection of path pairs on multicriteria ad hoc networks. Journal of Computer Networks and Communications.

[4] Sörensen K., Sevaux M., Glover F. (2018). Handbook of Heuristics.

[5] Trojovskỳ P., Dehghani M. (2022). Pelican optimization algorithm: a novel nature-inspired algorithm for engineering applications. Sensors.

[6] Suyanto S., Ariyanto A.A., Ariyanto A.F. (2022). Komodo mlipir algorithm. Appl. Soft Comput..

[7] Geem Z.W., Kim J.H., Loganathan G.V. (2001). A new heuristic optimization algorithm: harmony search. Simulation.

[8] Wang L., Yang R., Ni H., Ye W., Fei M., Pardalos P.M. (September 2015). A human learning optimization algorithm and its application to multi-dimensional knapsack problems. Appl. Soft Comput..

[9] Moosavi S.H.S., Bardsiri V.K. (November 2019). Poor and rich optimization algorithm: a new human-based and multi populations algorithm. Eng. Appl. Artif. Intell..

[10] Yang X.-S., Gandomi A.H. (July 2012). Bat algorithm: a novel approach for global engineering optimization. Eng. Comput..

[11] Mirjalili S., Mirjalili S.M., Lewis A. (2014). Grey wolf optimizer. Adv. Eng. Software.

[12] Mirjalili S., Lewis A. (2016). The whale optimization algorithm. Adv. Eng. Software.

[13] Yusup N., Zain A.M., Latib A.A. (March 2019). A review of Harmony Search algorithm-based feature selection method for classification. J. Phys. Conf..

[14] Abualigah L.M., Khader A.T., Al-Betar M.A. (2016). 2016 7th International Conference on Computer Science and Information Technology.

[15] Das S., Singh P.K., Bhowmik S., Sarkar R., Nasipuri M. (2016). A harmony search based wrapper feature selection method for holistic Bangla word recognition. Procedia Computer Science.

[16] Ramos C.C.O., Souza A.N., Chiachia G., Falcão A.X., Papa J.P. (November 2011). A novel algorithm for feature selection using Harmony Search and its application for non-technical losses detection. Comput. Electr. Eng..

[17] Yu S., Jia Y., Hu X., Ni H., Wang L. (2021). Intelligent Equipment, Robots, and Vehicles.

[18] Zhang P., Wang L., Du J., Fei Z., Ye S., Fei M., Pardalos P.M. (April 2022). Differential human learning optimization algorithm. Comput. Intell. Neurosci..

[19] Thirumoorthy K., Muneeswaran K. (February 2021). An elitism based self-adaptive multi-population Poor and Rich optimization algorithm for grouping similar documents. J. Ambient Intell. Hum. Comput..

[20] Nakamura R.Y.M., Pereira L.A.M., Rodrigues D., Costa K.A.P., Papa J.P., Yang X.-S. (2013). Swarm Intelligence and Bio-Inspired Computation.

[21] Jeyasingh S., Veluchamy M. (2017). Modified bat algorithm for feature selection with the Wisconsin diagnosis breast cancer (WDBC) dataset. Asian Pac. J. Cancer Prev. APJCP.

[22] Preeti, Kumar D. (October 2017). Feature selection for face recognition using DCT-PCA and Bat algorithm. Int. J. Inf. Technol..

[23] Al-Tashi Q., Rais H., Jadid S. (2018). Advances in Intelligent Systems and Computing.

[24] Hu P., Pan J.-S., Chu S.-C. (2020). Improved binary grey wolf optimizer and its application for feature selection. Knowl. Base Syst..

[25] Emary E., Zawbaa H.M., Hassanien A.E. (January 2016). Binary grey wolf optimization approaches for feature selection. Neurocomputing.

[26] Sharawi M., Zawbaa H.M., Emary E., Zawbaa H.M., Emary E. (2017). 2017 Ninth International Conference on Advanced Computational Intelligence.

[27] Zamani H., Nadimi-Shahraki M.-H. (2016). Feature selection based on whale optimization algorithm for diseases diagnosis. Int. J. Comput. Sci. Inf. Secur..

[28] Mafarja M., Mirjalili S. (January 2018). Whale optimization approaches for wrapper feature selection. Appl. Soft Comput..

[29] Bacanin N., Zivkovic M., Antonijevic M., Venkatachalam K., Lee J., Nam Y., Marjanovic M., Strumberger I., Abouhawwash M. (2023). Addressing feature selection and extreme learning machine tuning by diversity-oriented social network search: an application for phishing websites detection. Complex & Intelligent Systems.

[30] Sun L., Qin H., Przystupa K., Cui Y., Kochan O., Skowron M., Su J. (2022). A hybrid feature selection framework using improved sine cosine algorithm with metaheuristic techniques. Energies.

[31] Kareem S.S., Mostafa R.R., Hashim F.A., El-Bakry H.M. (2022). An effective feature selection model using hybrid metaheuristic algorithms for iot intrusion detection. Sensors.

[32] Zivkovic M., Stoean C., Chhabra A., Budimirovic N., Petrovic A., Bacanin N. (2022). Novel improved salp swarm algorithm: an application for feature selection. Sensors.

[33] Gharehchopogh F.S., Maleki I., Dizaji Z.A. (2022). Chaotic vortex search algorithm: metaheuristic algorithm for feature selection. Evolutionary Intelligence.

[34] Abdulkhaleq M.T., Rashid T.A., Alsadoon A., Hassan B.A., Mohammadi M., Abdullah J.M., Chhabra A., Ali S.L., Othman R.N., Hasan H.A., others (2022). Harmony search: current studies and uses on healthcare systems. Artif. Intell. Med..

[35] Bangyal W.H., Hameed A., Ahmad J., Nisar K., Haque M.R., Ibrahim A.A.A., Rodrigues J.J.P.C., Khan M.A., Rawat D.B., Etengu R. (2022). New modified controlled bat algorithm for numerical optimization problem. Comput. Mater. Continua (CMC).

[36] Akila S., Christe S.A. (2022). A wrapper based binary bat algorithm with greedy crossover for attribute selection. Expert Syst. Appl..

[37] Makhadmeh S.N., Alomari O.A., Mirjalili S., Al-Betar M.A., Elnagar A. (2022). Recent advances in multi-objective grey wolf optimizer, its versions and applications. Neural Comput. Appl..

[38] Nadimi-Shahraki M.H., Zamani H., Mirjalili S. (2022). Enhanced whale optimization algorithm for medical feature selection: a COVID-19 case study. Comput. Biol. Med..

[39] Priyadarshini J., Premalatha M., Čep R., Jayasudha M., Kalita K. (2023). Analyzing physics-inspired metaheuristic algorithms in feature selection with K-nearest-neighbor. Appl. Sci..

[40] Ganesh N., Shankar R., Čep R., Chakraborty S., Kalita K. (2023). Efficient feature selection using weighted superposition attraction optimization algorithm. Appl. Sci..

